# Comparison of alternative full and brief versions of functional status scales among older adults in China

**DOI:** 10.1371/journal.pone.0234698

**Published:** 2020-08-11

**Authors:** Jeremy Reich, Mark G. Thompson, Benjamin J. Cowling, A. Danielle Iuliano, Carolyn Greene, Yuyun Chen, Rachael Phadnis, Nancy H. L. Leung, Ying Song, Vicky J. Fang, Cuiling Xu, Qigang Dai, Jun Zhang, Hongjun Zhang, Fiona Havers, on behalf of the CARES investigators

**Affiliations:** 1 Influenza Division, Centers for Disease Control and Prevention, Atlanta, Georgia, United States of America; 2 WHO Collaborating Centre for Infectious Disease Epidemiology and Control, School of Public Health, Li Ka Shing Faculty of Medicine, The University of Hong Kong, Hong Kong Special Administrative Region, China; 3 Abt Associates, Cambridge, Massachusetts, United States of America; 4 Chinese National Influenza Center, National Institute for Viral Disease Control and Prevention, Collaboration Innovation Center for Diagnosis and Treatment of Infectious Diseases, Chinese Center for Disease Control and Prevention, Beijing, China; 5 Department of Acute Infectious Disease Control and Prevention, Jiangsu Provincial Center for Disease Control and Prevention, Nanjing, China; 6 Suzhou Center for Disease Prevention and Control, Suzhou, China; 7 Yancheng Center for Disease Prevention and Control, Yancheng, China; University of Malaya, MALAYSIA

## Abstract

**Background:**

Brief assessments of functional status for community-dwelling older adults are needed given expanded interest in the measurement of functional decline.

**Methods:**

As part of a 2015 prospective cohort study of older adults aged 60–89 years in Jiangsu Province, China, 1506 participants were randomly assigned to two groups; each group was administered one of two alternative 20-item versions of a scale to assess activities of daily living (ADL) and instrumental activities of daily living (IADL) drawn from multiple commonly-used scales. One version asked if they required help to perform activities (ADL-IADL-HELP-20), while the other version provided additional response options if activities could be done alone but with difficulty (ADL-IADL-DIFFICULTY-20). Item responses to both versions were compared using the binomial test for differences in proportion (with Wald 95% confidence interval [CI]). A brief 9-item scale (ADL-IADL-DIFFICULTY-9) was developed favoring items identified as difficult or requiring help by ≥4%, with low redundancy and/or residual correlations, and with significant correlations with age and other health indicators. We repeated assessment of the measurement properties of the brief scale in two subsequent samples of older adults in Hong Kong in 2016 (aged 70–79 years; n = 404) and 2017 (aged 65–82 years; n = 1854).

**Results:**

Asking if an activity can be done alone but with difficulty increased the proportion of participants reporting restriction on 9 of 20 items, for which 95% CI for difference scores did not overlap with zero; the proportion with at least one limitation increased from 28.6% to 34.2% or an absolute increase of 5.6% (95% CI = 0.9–10.3%), which was a relative increase of 19.6%. The brief ADL-IADL-DIFFICULTY-9 maintained excellent internal consistency (α = 0.93) and had similar ceiling effect (68.1%), invariant item ordering (H trans = .41; medium), and correlations with age and other health measures compared with the 20-item version. The brief scale performed similarly when subsequently administered to older adults in Hong Kong.

**Conclusions:**

Asking if tasks can be done alone but with difficulty can modestly reduce ceiling effects. It’s possible that the length of commonly-used scales can be reduced by over half if researchers are primarily interested in a summed indicator rather than an inventory of specific types of deficits.

## Introduction

The measurement of functional status is relevant to an expanding range of research topics within gerontology [1, 2] and vaccinology [3] due to associations with quality of life, healthcare costs, and clinical decision-making [4, 5]. Measures of functional status may be more closely associated with deterioration in immune function [6] than chronological age which has traditionally been used as a proxy for immunosenescence [7]. In evaluations of influenza vaccines, for example, broader measures of frailty that include limitations in functional status have been associated with antibody response to vaccination [8, 9] and differences in clinical protection from vaccination [10]. For studies such as these, where functional status is not the primary topic of interest, the ideal measurement tool should focus on functional capabilities and limitations that are relevant to community-dwelling older adults and should be brief, given budget and time constraints.

Developing valid, reliable, and efficient ways of assessing ADLs and IADLs has been a common challenge in geriatric research for decades. Early measures developed for institutionalized and disabled older adults had high ceiling effects when administered in community-based studies, with the vast majority of participants having almost perfect functioning and few deficits [11]. The Groningen Activity Restriction Scale (GARS) [12] and other measures specifically designed for community-dwelling older adults included physically and cognitively demanding activities that were more sensitive to modest impairment. The GARS and other measures interested in the early phases of decline and the subjective nature of some functional deficits [13, 14] also introduced new response options so participants can identify activities that could be performed without help but no longer with ease.

Here, we describe the results of four steps we took to develop and evaluate measures of functional status in the context of a prospective cohort study of older adults in Eastern China [15] and two ongoing influenza vaccine immunogenicity trials in Hong Kong [16]. Our first aim was to identify a functional status measurement tool that was appropriate for community-dwelling older adults in China. Specifically, we compared the measurement properties of two alternative versions of a 20-item scale to assess both activities of daily living (ADL) and instrumental activities of daily living (IADL) that were featured in commonly-used ADL-IADL scales and indices (described below). The two versions differed in their response options. Our local partners preferred to use a version that only asks older adults whether or not they require help to perform activities, similar to one application of GARS [12] and several other assessments of ADL-IADL [17, 18]. We compared this to a second version that includes both the “need help” options and the original GARS response options which ask the participant to identify activities that can be performed alone but with difficulty. As noted by the developers of GARS [19, 20] and other ADL and/or IADL tools [21, 22], we expected the version that included the full range of response options would be more sensitive to modest functional impairments and thus be more appropriate to studies of community-dwelling older adults. Nonetheless, to our knowledge, no previous study has compared the information value of these alternative response options.

Our second aim was to examine whether the number of ADL-IADL items could be reduced, while retaining items with high information value and maintaining high internal consistency. Third, we used insights from these comparisons to develop an ADL-IADL scale with fewer items and then compared results from this brief scale to other frequently used ADL-IADL scales. Fourth, we subsequently assessed whether the same pattern of findings regarding the performance of this brief scale was repeated two additional study populations.

## Materials and methods

### Study samples

This study includes data from three samples of older adults. The primary aims of this study were examined in the *China Ageing Respiratory Infections Study* (CARES) prospective cohort study of community-dwelling older adults 60–89 years of age residing in the cities of Suzhou and Yancheng in Jiangsu Province in Eastern China, in 2015. Most recruitment occurred through telephone and home visits to a random selection of older adults in the region; further details on methods and measures for CARES are described elsewhere [15]. The 1,506 CARES participants were randomly assigned to two groups; each group was administered one of two versions of the 20-item ADL-IADL scale.

For the final study aim, the brief scale developed through the analysis of information from the CARES sample was subsequently administered to community-dwelling participants in two influenza vaccine immunogenicity trials in Hong Kong: (a) *Immunogenicity of twice-annual vaccination against seasonal influenza for two hemispheres in older adults in Hong Kong–a randomized controlled trial* (RETAIN) in 2016 with 404 older adults aged 70–79 years recruited from ambulatory clinics, and (b) *Immunogenicity of alternative annual influenza vaccines in older adults in Hong Kong–a randomized controlled trial* (PIVOT) in 2017 with 1854 older adults aged 65–82 years recruited from senior centers and other community outreach efforts. In all three samples, older adults with clinical diagnoses of dementia and/or those who did not pass the Mini-cog dementia screen tool [23] were excluded.

### Measures

The 20-item ADL-IADL measure include activities featured in the GARS [19, 20, 24], the Barthel Index [22, 25], the Frailty Index [26], Katz ADL scale [17], the Groningen Frailty Indicator [27], the Lawton IADL Scale [28], the Vulnerable Elders Survey (VES) [29], and indicators developed from two large longitudinal studies of older adults in Canada [30] and Beijing, China [31]. During the development of ADL and IADL measures over the past 50 years, various indicators have borrowed and refined the activities that are measured and how these items are phrased. Eighteen of the 20 ADL-IADL items we examined were featured activities in the GARS, though the exact wording differed for 11 of these items (S1 Table of S1 File). Item wordings were changed to improve consistency with how these activities were featured in other scales and/or to improve comprehension when translated based on feedback from study staff and pilot participants. One activity was only featured in the GARS, but all other activities were included in 2 to 7 measures of the nine measures we reviewed (S1 Table of S1 File). The current measure includes 12 activities focused on ADL and 8 activities focused on IADL, including ambulation, shopping, personal hygiene, food preparation, laundry, housekeeping, dressing, and feeding (S1 Table of S1 File).

The measures were administered by trained study staff. Half of the CARES participants (n = 748) were randomly assigned to receive the ADL-IADL scale version that asked if each activity could be done alone without help or if the activity required help sometimes, often, very often, or all the time (ADL-IADL-HELP-20). The other half (n = 758) received a version with the same 20 items, the same options for needing help, but if the activity could be performed alone, additional response options were given to distinguish between activities that could be done alone but with great difficulty, some difficulty, or no difficulty (ADL-IADL-DIFFICULTY-20). This group of participants were also asked questions that could be used to create scores that were similar to two other commonly used brief functional status measures: (1) the Vulnerable Elders Survey (VES) [29] is a 13-item scale that includes age, self-rated health, difficulty with activities, and specific physical capabilities (e.g., walking a quarter mile, carrying objects as heavy as 10 pounds), and (2) the Lawton IADL scale [28] rates level of performance on 8 activities (e.g., use of telephone, medication use, handling finances). The published scoring system was applied for both scales, though the exact wording of some activities differed from the original scales due to Chinese translation and the need for common administration methods.

A common set of additional variables were assessed in the CARES cohort and in the PIVOT and RETAIN trials (S2 Table of S1 File), including socio-demographic characteristics (age, sex, marital status, and educational level), self-reported days sick and days healthy in the past month, self-rated current health status [32], and number of days leaving home in the prior week. The number of chronic conditions (including cardiovascular, lung, kidney, liver, or neurological diseases; diabetes, cancers, osteoarthritis, or depression) was assessed in CARES and PIVOT, but not RETAIN. Additional variables, such as self-reported number of falls in the past 12 months, number of hospitalizations in the past two years, number of medications taken each day, and the Mini-Mental State Examination (MMSE) [33], which assesses cognitive functioning, were only assessed in the CARES cohort [33] (S2 Table of S1 File).

### Statistical analyses

Similar to previous efforts to develop and evaluate functional status scales or sub-scales [27, 34], we used a combination of traditional psychometric evaluations (classical test theory) and more recent psychometric evaluations (item response theory). For the first aim, we compared the proportion of participants reporting limitations on an item-by-item basis forADL-IADL-HELP-20 andADL-IADL-DIFFICULTY-20, using a binomial test for differences in proportion with Wald 95% confidence interval (CI). We also compared the total proportion of older adults reporting at least one limitation across items using the same statistic. For the total summed scales, additional comparisons included: (a) internal consistency using Cronbach’s alpha (α), with standard thresholds [35], (b) invariant item ordering [36], using H trans (H^T^) and previously published thresholds [37, 38], and (c) correlations with other health-related variables as indicators of concurrent validity. All scale and item scores were log-transformed prior to assessing correlations given the skewed nature of response distributions.

**Fig 1:**
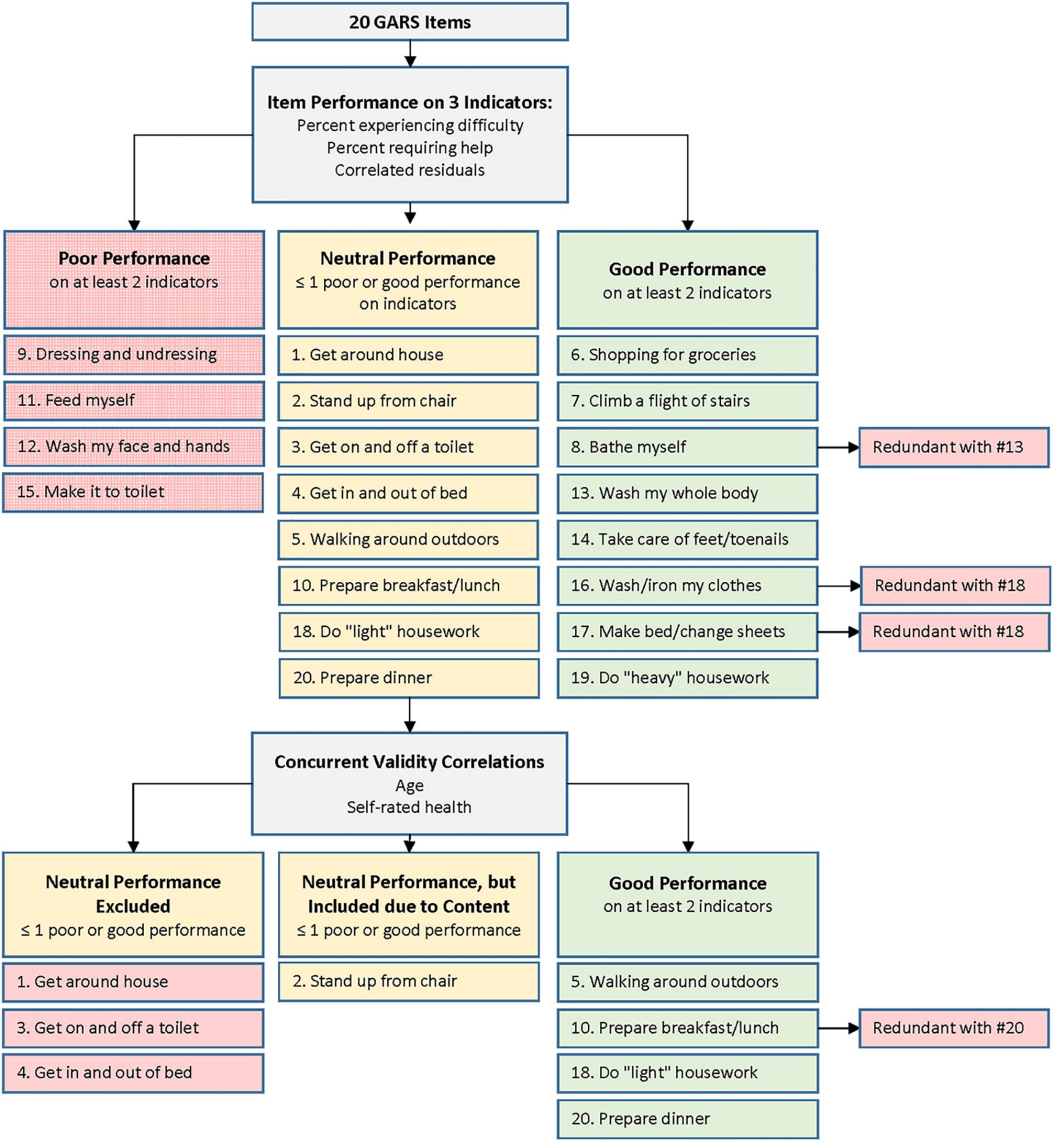
Decision tree of ADL-IADL item categorization into good, neutral, and poor performing item categories.

For our second aim we identified ADL-IADL items that could potentially be eliminated using a two-stage process (Fig 1). In the first stage, items were considered to have good information value if they identified a substantial percentage of participants (≥4%) as having limitations; items were considered to have low information value if ≤2% of participants had limitations. Also, in this first stage, a principal component factor analysis (with oblique rotation) confirmed that a single common factor explained 65% of the variance among the ADL-IADL-DIFFICULTY-20 items. We calculated the correlation of each item with the total ADL-IADL-DIFFICULTY-20 scale after adjusting for the common factor score. Items with relatively low residual correlations (<1 standard deviation [SD] below the mean correlation) were considered good performers because most of the item variance was explained by the common factor; items with relatively high residual correlations (>1 SD above the mean) were considered poor performers because a substantial amount of item variance was not explained by the common factor; this approach is consistent with previous studies on measurement development [34, 39]. In the second stage, items with neutral performance in the first stage were evaluated based on indicators of concurrent validity; specifically, we examined Pearson correlations with age, self-rated health, and number of days per week the participant leaves the home. In both stages one and two, items with redundant content were also excluded if a better performing item focused on a very similar task.

For our third aim, the resulting brief scale (ADL-IADL-DIFFICULTY-9) was compared to the full ADL-IADL-DIFFICULTY-20 and two other previously published scales (the VES and Lawton IADL scale) based on internal consistency (Cronbach’s alpha), invariant item ordering (using H^T^), and concurrent validity (correlations with other health indicators). Fourth, we examined external validity by evaluating these same indicators when the ADL-IADL-DIFFICULTY-9 scale was subsequently administered in the RETAIN and PIVOT samples in Hong Kong.

The study protocol received ethical approval from the Institutional Review Board of the University of Hong Kong (Ref: UW15 404), and the Ethics Committee of Jiangsu Provincial Center for Disease Control and Prevention (Ref: JSJK2015-B013-02). Written consent was provided by all participants.

## Results

### Participant characteristics

Demographic and health characteristics of participants in the three study samples are presented in Table 1 (additional descriptive information in S3 Table of S1 File). The random sub-samples that were administered the two ADL-IADL scale versions had similar characteristics, with the exception of somewhat better self-reported health among those who were administered the ADL-IADL-HELP-20. By study design, the age composition differs for CARES (aged 60–89 years), RETAIN (aged 70–79 years), and PIVOT (aged 65–82 years). Women comprised over half of the participants in CARES (55%) and PIVOT (57%), but only 34% of RETAIN participants. About two-thirds of participants had secondary schooling or higher education in the Hong Kong-based RETAIN (62%) and PIVOT (65%) cohorts compared to only 15% of the CARES participants in mainland China. The RETAIN and PIVOT participants had worse self-rated health than CARES participants. However, the three samples were similar in numbers of sick and healthy days reported in the past month.

**Table 1:** Sample characteristics in CARES cohort and RETAIN and PIVOT vaccine trrials.

Study Name	CARES		CARES		RETAIN		PIVOT	
Scale	ADL-IADL-HELP-20		ADL-IADL-DIFFICULTY-20		ADL-IADL-DIFFICULTY-9		ADL-IADL-DIFFICULTY-9	
Characteristics, No. (Percent)	N (748)	(%)	N (758)	(%)	N (404)	(%)	N (1854)	(%)
Age ^a^								
60 to 64	122	(16)	120	(16)	NM		NM	
65 to 69	154	(21)	142	(19)	NM		795	(43)
70 to 74	148	(20)	153	(20)	210	(52)	507	(27)
75 to 79	105	(14)	104	(14)	194	(48)	379	(20)
80 to 84	173	(23)	185	(24)	NM		173	(9)
85 to 89	46	(6)	54	(7)	NM		NM	
Sex								
Male	334	(45)	337	(44)	266	(66)	726	(39)
Female	414	(55)	421	(56)	138	(34)	1128	(61)
Marital status								
Not married	237	(32)	254	(33)	82	(20)	515	(28)
Married	511	(68)	504	(67)	322	(79.7)	1339	(72)
Educational attainment								
Secondary schooling or above	115	(15)	114	(15)	250	(62)	1069	(58)
Less	633	(85)	644	(85)	154	(38)	785	(42)
Self-rated health *								
Poor-Fair	157	(21)	212	(28)	238	(59)	969	(52)
Good	284	(38)	294	(39)	109	(27)	566	(31)
Very Good-Excellent	307	(41)	252	(33)	57	(14)	319	(17)
History of falling in prior year								
Yes	67	(9)	71	(9)	NM		NM	
No	680	(91)	687	(91)	NM		NM	
**Health Status, Mean (SD)**								
Chronic conditions (total count)	1.0	(1.0)	1.0	(1.0)	NM		1.3	(1.1)
Hospitalizations (in 2 years)	0.2	(0.6)	0.2	(0.6)	NM		NM	
Medications per day (total count)	0.9	(1.2)	0.9	(1.2)	NM		NM	
Mini-Mental State Examination	25.0	(3.6)	24.9	(3.7)	NM		NM	
Poor health days (in past month)	NM		2.5	(6.0)	3.0	(7.5)	2.2	(5.9)
Sick in bed days (in past month)	NM		0.7	(2.9)	NM		0.7	(3.6)
Good health days (in past month)	NM		20.1	(11.0)	17.1	(13.2)	21.5	(13.7)
Days leaving home (in prior week)	6.5	(1.5)	6.6	(1.4)	NM		6.6	(1.2)

Abbreviations: NM indicates that variable was not measured in sample; CARES is the prospective cohort study in Eastern China; PIVOT and RETAIN are vaccine trials in Hong Kong; ADL is activities of daily living; IADL is instrumental ADL

^a^ Ages for enrolled participants were ≥60 years for CARES, 70–79 for RETAIN, and 65 to 82 years for PIVOT

* Self-rated health rated from poor (1) to excellent (5) is significantly different (p < 0.001) between the CARES sub-samples and between CARES and the two vaccine trial samples.

### Comparison of the two ADL-IADL scale response option versions

When the definition of functional limitation was expanded from tasks that require help (ADL-IADL-HELP-20) to include tasks that can be performed alone but with difficulty (ADL-IADL-DIFFICULTY-20), the proportion of participants with limitations increased for all task items; this increase was statistically significant for 9 of the 20 items (Table 2). For example, the numbers of participants that reported limitations performing light and heavy housework or climbing stairs were statistically significantly higher when participants were asked if they could do the task alone but found it difficult. As a summed scale, ADL-IADL-HELP-20 identified 28.6% of participants as having at least one limitation compared to 34.2% using the ADL-IADL-DIFFICULTY-20. Thus, asking if an activity can be done alone but with difficulty increased the proportion of cohort participants with at least one limitation by 5.6% (95% CI = 0.9–10.3%); this is a relative increase of 19.6%.

**Table 2:** Percentage (%) of participants reporting any functional limitation on scales with response options that focused on need for help only (ADL-IADL-HELP-20) and response options also asessing difficulty completing tasks without help (ADL-IADL-20-DIFFICULTY) in the CARES Cohort in Eastern China, 2015.

Scale		ADL-IADL-HELP-20		ADL-IADL-DIFFICULTY-20		
Response options dichotomy		Requiring *any* help		Requiring *any* help or experiencing *any* difficulty		
		Percent	(SD)	Percent	(SD)	Difference of Proportions (95% CI) ^a^
1)	**Get around from room to room in my house**	1.2%	(0.11)	2.9%	(0.17)	**1.7% (0.3, 3.1%)**
2)	**Stand up from sitting in a chair**	1.9%	(0.14)	4.2%	(0.20)	**2.4% (0.6, 4.1%)**
3)	**Get on and off a toilet**	1.1%	(0.10)	2.9%	(0.17)	**1.8% (0.4, 3.2%)**
4)	**Get in and out of bed**	1.5%	(0.12)	3.2%	(0.18)	**1.7% (0.2, 3.2%)**
5)	**Walking around outdoors or in my neighborhood**	2.3%	(0.15)	5.0%	(0.22)	**2.7% (0.9, 4.6%)**
6)	Shopping for groceries	7.1%	(0.26)	9.4%	(0.29)	2.3% (-0.5, 5.1%)
7)	**Climb a flight of stairs**	15.1%	(0.36)	21.0%	(0.41)	**5.9% (0.2, 9.7%)**
8)	Bathe myself in a shower or bath tub	6.4%	(0.25)	8.5%	(0.28)	2.0% (-0.6, 4.7%)
9)	Dressing and undressing	2.0%	(0.14)	2.9%	(0.17)	0.9% (-0.7, 2.5%)
10)	Prepare my breakfast or lunch	4.7%	(0.21)	5.3%	(0.22)	0.6% (-1.6, 2.8%)
11)	Feed myself	0.7%	(0.08)	1.1%	(0.10)	0.4% (-0.6, 1.3%)
12)	Wash my face and hands	0.9%	(0.10)	1.2%	(0.11)	0.3% (-0.8, 1.3%)
13)	Wash my whole body by taking a shower or bath	6.3%	(0.24)	7.5%	(0.26)	1.3% (-1.3, 3.8%)
14)	Take care of my feet and toenails	8.6%	(0.28)	11.4%	(0.32)	2.8% (-0.2, 5.8%)
15)	**Make it to the toilet without an accident**	1.3%	(0.11)	2.9%	(0.17)	**1.6% (0.1, 3.0%)**
16)	Wash and iron my clothes	9.9%	(0.30)	10.8%	(0.31)	0.9% (-2.1, 0.4%
17)	Make the bed or change sheets	6.6%	(0.25)	9.1%	(0.29)	2.6% (-0.2, 5.3%)
18)	**Do "light" housework like dusting or tidying up**	4.3%	(0.20)	7.5%	(0.26)	**3.3% (0.9, 5.6%)**
19)	**Do "heavy" housework like mopping or vacuuming**	20.9%	(0.41)	25.1%	(0.43)	**4.2% (0.0, 8.5%)**
20)	Prepare dinner	7.2%	(0.26)	8.2%	(0.27)	1.0% (-1.7, 3.7%)
**Any functional limitation for at least 1 item**		28.6%	(0.45)	34.2%	(0.47)	**5.6% (0.9, 10.3%)**

Abbrviations: CARES is the prospective cohort study in Eastern China; ADL is activities of daily living; IADL is instrumental ADL.

^a^ Test for differences in proportions assesses equality of proportions between two independent samples. Wald 95% confidence interval (CI) that does not overlap with 0 indicates statistical significance and is bolded.

The item and scale performance indicators were similar for the scale with either response option versions (S4 Table of S1 File). Both scales have excellent internal consistency (α ≥ 0.90). Invariant item ordering was high (H^T^ ≥ 0.5) for ADL-IADL-HELP-20 and medium for ADL-IADL-DIFFICULTY-20 (0.4 ≤ H^T^ < 0.5). Both versions were similarly associated with age, self-rated health, chronic conditions, MMSE scores, and number of days leaving home per week. The only statistically significant difference observed was a correlation of 0.16 (95% CI = 0.09, 0.23) between ADL-IADL-DIFFICULTY-20 and number of falls in the past year compared to a zero association (95% CI = -0.07, 0.08) with ADL-IADL-HELP-20.

### Selection of nine items for a brief ADL-IADL-DIFFICULTY scale

As summarized in Fig 1, the number of ADL-IADL-DIFFICULTY-20 items was reduced in two stages (S5 Table of S1 File). Based on item performance, four items were excluded as poor performers because few older adults (≤2%) reported needing help or experiencing difficulty and/or the item had high residual correlations with other items. Eight items displayed good performance on these indicators; of these, three items were excluded due to redundant content with other good performing items, leaving five items with good performance. Eight items with neutral performance in this first stage were then examined for concurrent validity. Four items were associated with at least two of three indicators (age, self-rated health, and days leaving home per week); of these, one item was excluded due to redundant content. Four items were related to one indicator, and one of these (standing up from chair) was retained because it addresses a mobility task that is not covered by other items. The nine selected items are referred to as ADL-IADL-DIFFICULTY-9 include ambulating, shopping, personal hygiene, housekeeping, and food preparation activities:

- Walking around outdoors or in my neighborhood
- Shopping for groceries
- Climb a flight of stairs
- Wash my whole body by taking a shower or bath
- Take care of my feet and toenails
- Do "light" housework like dusting or tidying up
- Do "heavy" housework like mopping or vacuuming the floor
- Prepare dinner
- Stand up from sitting in a chair

### Comparison of ADL-IADL-DIFFICULTY-9 scale with other scales

The brief version (ADL-IADL-DIFFICULTY-9) maintained excellent internal consistency (α = 0.93) and was similar to the 20-item version in invariant item ordering and ceiling effect (Table 3). The ADL-IADL-DIFFICULTY-9 has better internal consistency than the VES (α = 0.75) and Lawton IADL (α = 0.73) scales that were administered to this same sub-sample (Table 3). However, the VES and Lawton had better invariant item ordering ratings indicating that the ranking from most to least endorsed items within these scales was more consistent than the ranking for ADL-IADL-DIFFICULTY-9. The magnitude of correlations between the ADL-IADL-DIFFICULTY-9 and other health and functioning indicators were similar to those observed for the longer ADL-IADL-DIFFICULTY-20, VES, and Lawton IALD scales (Table 3).

**Table 3:** Measurement indicators and concurrent validity indicators for ADL-IADL-DIFFICULTY-9, ADL-IADL-DIFFICULTY-20, and two other published functional status measures.

Scale	ADL-IADL-DIFFICULTY-9		ADL-IADL-DIFFICULTY-20		Similar to VES ^a^		Similar to Lawton IADL ^a^	
N	758		758		758		758	
**Measurement Indicators**								
Cronbach's Alpha ^b^	0.93 (Excellent)		0.95 (Excellent)		0.64 (Marginal)		0.73 (Acceptable)	
Invariant Item Ordering H^T^ ^c^	0.41 (Medium)		0.45 (Medium)		0.75 (High)		0.99 (High)	
Ceiling Effect	68.1%	(64.6, 71.3%)	65.8%	(62.3, 69.2%)	Not measured ^d^		83.3%	(80.4, 85.8%)
**Correlation to Scale Total** ^e^								
Age	**0.34**	**(0.28, 0.40)**	**0.30**	**(0.23, 0.36)**	**0.16**	**(0.09, 0.23)**	**0.27**	**(0.20, 0.33)**
Self-rated health	**-0.29**	**(-0.35, -0.22)**	**-0.27**	**(-0.34, -0.21)**	**-0.18**	**(-0.24, -0.11)**	**-0.21**	**(-0.28, -0.15)**
Number of falls (prior year)	**0.18**	**(0.11, 0.25)**	**0.16**	**(0.09, 0.23)**	0.02	(-0.05, 0.10)	**0.10**	**(0.03, 0.17)**
Hospitalizations (in 2 years)	0.04	(-0.03, 0.11)	0.04	(-0.03, 0.11)	0.02	(-0.06, 0.09)	0.03	(-0.04, 0.10)
Medications per day (total count)	**0.07**	**(0.00, 0.14)**	0.07	(-0.00, 0.14)	**0.09**	**(0.02, 0.16)**	**0.08**	**(0.01, 0.15)**
Chronic conditions (total count)	**0.16**	**(0.09, 0.23)**	**0.15**	**(0.08, 0.22)**	**0.15**	**(0.08, 0.21)**	**0.14**	**(0.07, 0.21)**
Mini-Mental State Examination	**-0.29**	**(-0.35, -0.22)**	**-0.27**	**(-0.33, -0.20)**	**-0.22**	**(-0.29, -0.15)**	**-0.22**	**(-0.28, -0.15)**
Poor health days (in past month)	**0.16**	**(0.09, 0.23)**	**0.19**	**(0.12, 0.26)**	**0.18**	**(0.11, 0.24)**	**0.17**	**(0.10, 0.24)**
Sick in bed days (in past month)	**0.24**	**(0.17, 0.30)**	**0.28**	**(0.21, 0.34)**	**0.22**	**(0.16, 0.29)**	**0.19**	**(0.12, 0.26)**
Good health days (in past month)	-0.07	(-0.14, 0.01)	**-0.09**	**(-0.16, -0.02)**	**-0.25**	**(-0.32, -0.19)**	**-0.09**	**(-0.16, -0.02)**
Days leaving home (in prior week)	**-0.30**	**(-0.37, -0.24)**	**-0.30**	**(-0.36, -0.23)**	**-0.30**	**(-0.37, -0.24)**	**-0.21**	**(-0.28, -0.14)**

Pearson correlations performed using the summed score of all item responses; significant correlations are bolded

Abbreviations: DL is activities of daily living; IADL is instrumental ADL; Vulnerable Elders Survey (VES); Lawton Instrumental Activities of Daily Living Scale (Lawton IADL)

^a^ The published scoring system was applied for both scales, though the exact wording of some activities differed from the original scales due to Chinese translation and the need for common administration methods

^b^ Categorical descriptions are based on previously published conventions for Cronbach’s alpha

^c^ Using previously establised conventions for H^T^, items marked for good performance when mean Invariant Item Ordering (IIO) score is ≥0.50 and flagged for poor performance when IIO score is ≤0.10

^d^ VES scale can not have ceiling effect measured by nature of questionnaire design

^e^ All scale and item scores were log-transformed prior to assessing correlations given the skewed nature of almost all distributions

### Performance of ADL-IADL-DIFFICULTY-9 scale in two subsequent studies

The ADL-IADL-DIFFICULTY-9 items were administered in two subsequent studies in Hong Kong. To aid in cross-study comparisons, Table 4 presents the measurement indicators with CARES participants narrowed to similar age ranges for these studies. The ADL-IADL-DIFFICULTY-9 maintained good (α = 0.82) to excellent (α = 0.90) internal consistency in the RETAIN and PIVOT samples, respectively. The ceiling effect of the summed scale was also statistically similar in the new samples. However, the invariant item ordering ratings were lower in the new samples compared to its performance with similar age groups in the CARES sample; in other words, consistency in which activities were reported as most to least difficult were lower among RETAIN and PIVOT participants. The pattern and direction of correlations between the ADL-IADL-DIFFICULTY-9 scores and other health indicators was similar for CARES and the new samples, with one exception. The ADL-IADL-DIFFICULTY-9 was not associated with the number of healthy days in the past month in the CARES (aged 65–84 years) sample, but this association was statistically significant among the PIVOT participants.

**Table 4:** Cross-study comparison of ADL-IADL-DIFFICULTY-9 from the CARES cohort and RETAIN and PIVOT vaccine trials.

Study name	CARES		RETAIN		CARES		PIVOT	
Age Group	70–79		70–79		65–84		65–82	
Scale Version	ADL-IADL-DIFFICULTY-9		ADL-IADL-DIFFICULTY-9		ADL-IADL-DIFFICULTY-9		ADL-IADL-DIFFICULTY-9	
N	257		404		584		1854	
**Measurement Indicators**								
Cronbach’s Alpha ^a^	0.95 (Excellent)		0.82 (Good)		0.94 (Excellent)		0.90 (Excellent)	
Invariant Item Ordering H^T^ ^b^	0.38 (Low)		0.24 (Inaccurate)		0.40 (Medium)		0.31 (Low)	
Ceiling Effect	68.5%	(62.4, 74.1%)	69.8%	(65.1, 74.2%)	65.9%	(61.9, 69.8%)	64.5%	(62.5, 66.5%)
**Correlation to Scale Total**								
Age	**0.16**	**(0.04, 0.28)**	**0.11**	**(0.02, 0.21)**	**0.27**	**(0.20, 0.35)**	**0.15**	**(0.09, 0.16)**
Self-rated health	**-0.24**	**(-0.35, -0.12)**	**-0.18**	**(-0.27, -0.09)**	**-0.27**	**(-0.34, -0.19)**	**-0.16**	**(-0.20, -0.12)**
Chronic conditions (total count)	**0.28**	**(0.16, 0.39)**	**0.27**	**(0.17, 0.36)**	**0.18**	**(0.10, 0.25)**	**0.21**	**(0.17, 0.25)**
Poor health days (in past month)	**0.17**	**(0.05, 0.29)**	**0.17**	**(0.07, 0.26)**	**0.15**	**(0.07, 0.23)**	**0.10**	**(0.06, 0.14)**
Sick in bed days (in past month)	**0.17**	**(0.05, 0.29)**	NM		**0.15**	**(0.7, 0.23)**	**0.15**	**(0.11, 0.19)**
Good health days (in past month)	-0.07	(-0.19, 0.06)	-0.07	(-0.21, 0.07)	-0.01	(-0.09, 0.07)	**-0.13**	**(-0.17, -0.09)**
Days leaving home (in prior week)	**-0.26**	**(-0.37, -0.14)**	NM		**-0.20**	**(-0.27, -0.12)**	**-0.15**	**(-0.19, -0.11)**

NM indicates that variable was not measured in sample

Pearson correlations performed using the summed score of all item responses

^a^ Categorical descriptions are based on previously published conventions for Cronbach’s alpha

^b^ Using previously establised conventions for H^T^, items marked for good performance when mean Invariant Item Ordering (IIO) score is ≥0.50 and flagged for poor performance when IIO score is ≤0.10

^c^ All scale and item scores were log-transformed prior to assessing correlations given the skewed nature of almost all distributions

## Discussion

We compared the measurement properties of two versions of an ADL and IADL measure (with activities drawn from commonly used scales) in the CARES cohort of community-dwelling older adults aged 60–89 years in Jiangsu Province, China, and developed a brief 9-item version of the scale that we further examined in two subsequent studies. Functional deficits were identified in about 20% more of the participants who were asked about difficulty performing activities alone (ADL-IADL-DIFFICULTY-20) compared to the other random half of participants who were only asked about needing help from others to perform activities (ADL-IADL-HELP-20). The brief scale we introduced here (ADL-IADL-DIFFICULTY-9) maintained excellent internal consistency and had almost identical associations with age and other measures of health and functioning when compared to the full 20-item version in the CARES cohort and when the brief scale was administered to older adults in Hong Kong.

To our knowledge, no prior study had compared the added value of response options that considered activities that could be done independently but with difficulty. We found that the expanded response options on ADL-IADL-DIFFICULTY-20 increased the proportion of older adults reporting a deficit on about half (9 of 20) of the items. This was most notable for the more physically demanding activities, such as “heavy housework” and “climb a flight of stairs.” Nonetheless, even with the expanded response options, two-thirds of CARES participants (65.8%) reported requiring no help and having no difficulty with ADLs and IADLs. Thus, assessments that include even more physically, cognitively, and socially challenging tasks (e.g., [34, 40]) may be needed to further reduce the ceiling effect.

Our findings suggest that the length of ADL-IADL scales like the GARS can be reduced by over half if researchers are primarily interested in a summed indicator rather than an inventory of specific types of deficits. The brief version (ADL-IADL-DIFFICULTY-9) had a similar ceiling effect (68.1%) to the 20-item version, in part because item selection favored activities that required help and/or were difficult to perform alone by at least 4% of participants. The retained items included activities that required mobility (e.g., walk around outdoors), physical stamina (e.g., climb a flight of stairs), dexterity (e.g., take care of my feet and toenails), and more complex mental and physical coordination (e.g., do “heavy” housework). One reason for excluding items was redundancy with other items. Although identifying specific deficits is helpful in clinical care and occupational therapy, multiple items regarding different types of housework, for example, do not offer added information in a summed scale. Other items were excluded because few participants found these difficult, which is to be expected since these activities are typically essential for independent living in the community (e.g., dressing and undressing; feed myself).

Nonetheless, the exclusion of 11 items had almost no impact on the internal consistency of the summed scale, which remained excellent (α >.90) for the brief ADL-IADL-DIFFICULTY-9, and notably higher than two other widely used ADL and IADL measures, VES (α = .64) and Lawton IADL (α = .73), which were administered to the same participants. Associations with indicators of criterion validity were also largely unchanged for the brief scale, since we intentionally selected items associated with age, self-rated health, and the number of days the participant left home in the prior week. Nonetheless, the pattern and magnitude of associations between the brief scale and health indicators were also similar to those of the VES and Lawton IADL. Results from the administration of the ADL-IADL-DIFFICULTY-9 in two new studies in Hong Kong were reassuring; the ceiling effect, internal consistency, and pattern of associations were similar, despite differences in sex, age, education, culture, and environments of participants in Hong Kong versus Eastern China.

Among the study’s strengths are its comparison of two random samples of older adults who received alternative versions of response options. Our study also assessed the performance of our brief scale in two new samples independent of the original sample. Our examination of functional status in the context of other health indicators (including chronic conditions and medications, cognitive functioning, history of falls, and reports of health and illness in the prior month) and other ADL/IADL scales is also a strength. Our study contributes to a relatively small but growing literature on functional status and frailty measurement among older adults in China (e.g., [5, 31, 41, 42]), where the rapidly aging population presents numerous societal challenges [43]. Data on older adults in Eastern China, where CARES took place, is especially limited.

Our study also has at least three limitations. First, our assessment of ADL, IADL, other health indicators relied on self-report, which is likely subject to information biases, including errors in recall and social desirability bias. This likely resulted in under-reporting of impairment and increased measurement error. Similarly, the presence and extent of “difficulty” performing an activity requires subjective interpretation, which also introduces measurement error. Second, our findings are based on cross-sectional associations, and future research is needed to examine whether the full and brief scales have similar predictive associations with changes in health status over time. Third, we relied primarily on classical or traditional measurement approaches and did not fully take advantage of methods from item response theory (see [1, 27]). Some of our decision criteria, such as what constituted low percentage of responses (≤2%) were informed by the findings in this particular study and do not correspond to well-established measurement design rules. The “medium” level of invariant item ordering observed for the ADL-IADL-DIFFICULTY-9 and 20-item versions suggests the item hierarchies (or which activities were most and least difficult) varied across CARES participants; consistency was even worse in the Hong Kong samples. It is possible that further calibration of item difficulty and personal ability (e.g., with Rasch analysis [44]) might refine item selection or inform alternative approaches to scoring item responses. Nonetheless, high invariant item ordering is a challenging and rarely met measurement yardstick [34]. It is also possible that individual differences in which ADLs and IADLs are difficult to perform should be expected, especially in populations with wide variations in education, living arrangements, and proximity to family support [20].

As interest in measuring functional status continues to expand, brief scales like ADL-IADL-DIFFICULTY-9 may be useful for studies that wish to identify relatively modest or early signs of functional decline but wish to limit the time burden on participants or have other logistical or budget constraints. Since functional status is only one element of a multi-component frailty phenotype, it is often measured with only a few items in frailty scales [2, 26]. It’s possible that a brief functional status scale, such as the one presented here, could be incorporated into frailty measurement as a unique component alongside other assessments of self-reported health or performance-based measures [26, 45], which taken together may increase the ability to predict subsequent health outcomes, like falls [46], hospital admissions [4], and mortality [2, 4, 31].

## Supporting information

S1 File (PDF)

S1 Data (ZIP)
