# Supplementary material for: Comparison of alternative full and brief versions of functional status scales among older adults in China
Source: PLoS One. 2020 Aug 11;15(8):e0234698. doi: 10.1371/journal.pone.0234698 (PMC7418957; doi:10.1371/journal.pone.0234698)
Supplement: S1 File — (PDF) [file pone.0234698.s001.pdf]

Supplemental Table A. Functional status items and similarity to items in the Groningen Activity Restriction Scale and eight other scales or measures.

		GARS			Frailty Index	GFI	Lawton IADL	Barthel Index	Katz ADL	VES	Dementia	Beijing Aging	
Item No.	Current Study Items	Similar content	Same wording	GARS Item Phrasing	Similar Content and Phrasing								Number of Scales per Activity
1	Get around from room to room in my house	X		Can you, fully independently, get around in the house (if necessary, with a cane)?	X			X				X	**4**
2	Stand up from sitting in a chair	X	X	Can you, fully independently, stand up from sitting in a chair?				X				X	**3**
3	Get on and off a toilet	X	X	Can you, fully independently, get on and off the toilet?	X	X		X			X		**6**
4	Get in and out of bed	X	X	Can you, fully independently, get in and out of bed?				X			X	X	**4**
5	Walking around outdoors or in my neighborhood	X		Can you, fully independently, walk outdoors (if necessary, with a cane)?	X	X							**3**
6	Shopping for groceries	X		Can you, fully independently, do the shopping?		X	X	X		X	X	X	**7**
7	Climb a flight of stairs	X		Can you, fully independently, go up and down the stairs?	X			X				X	**3**
8	Bathe myself in a shower or bath tub				X			X	X	X	X	X	**7**
9	Dressing and undressing	X		Can you, fully independently, dress yourself?	X	X			X		X	X	**5**
10	Prepare my breakfast or lunch	X		Can you, fully independently, prepare breakfast or lunch?			X					X	**3**
11	Feed myself	X	X	Can you, fully independently, feed yourself?	X			X	X		X	X	**6**
12	Wash my face and hands	X	X	Can you, fully independently, wash your face and hands?				X					**2**
13	Wash my whole body by taking a shower or bath	X		Can you, fully independently, wash and dry your whole body?					X				**2**
14	Take care of my feet and toenails	X	X	Can you, fully independently, take care of your feet and toenails?							X		**2**
15	Make it to the toilet without an accident, like losing control of urination or bowel movement							X	X			X	**4**
16	Wash and iron my clothes	X	X	Can you, fully independently, wash and iron your clothes?			X						**2**
17	Make the beds or change sheets	X		Can you, fully independently, make the beds?									**1**

18	Do “light” housework like dusting or tidying up	X	X	Can you, fully independently, do "light" household activities (for example dusting and tidying up)?			X			X			**3**
19	Do “heavy” housework like mopping or vacuuming the floor	X		Can you, fully independently, do "heavy" household activities (for example, mopping, cleaning the windows, and vacuuming)?			X			X			**3**
20	Prepare dinner	X	X	Can you, fully independently, prepare dinner?			X					X	**3**
**Number of activities featured in each scale:**		**18**	**9**		**7**	**4**		**10**	**5**	**4**	**7**	**11**	

Abbreviations and citations:

GARS: Groningen Activity Restriction Scale. (1) Saumeijer et al. The Groningen Activity Restriction Scale for Measuring Disability: Its utility in International Comparisons. Am J Public Health 1994, 84 (8) 1270-1273. (2) Kempen et al. The assessment of disability with the Groningen Activity Restriction Scale: Conceptual framework and psychometric properties. Soc Sci Med 1996, 43 (11): 1601-1610.

Frailty Index: Searle et al. A standard procedure for creating a frailty index. BMC Geriatrics 2008, 8: 24

GFI: Groningen Frailty Indicator. Bielderman et al.: Multidimensional structure of the Groningen Frailty Indicator in community-dwelling older people. BMC Geriatrics 2013 13:86.

Lawton IADL: The Lawton Instrumental Activities of Daily Living Scale. Lawton & Brody. Assessment of older people: Self-maintaining and instrumental activities of daily living. The Gerontologist 1969; 9(3): 179-186.

Barthel Index. Wake & Hewer. Functional abilities after stroke: measurement, natural history and progression. J Neurol Neurosurg Psychiatry 1987; 50(2): 177-82.

Katz ADL: Katz Activities of Daily Living. Katz et al. Studies of illness in the aged. The index of ADL: A standardized measure of biological and psychosocial function. JAMA 1963, 185: 914-919

VES: Vulnerable Elders Survey. Saliba et al. The Vulnerable Elders Survey: A tool for identifying vulnerable older people in the community. Geriatrics 2001, 49(12): 1691

Dementia: Song et al. Age-related deficit accumulation and the risk of late-life dementia. Alzheimer's Research & Therapy 2014, 6: 54.

Beijing Aging: Beijing Longitudinal Study of Aging. Shi et al. Analysis of frailty and survival from late middle age in the Beijing Longitudinal Study of Aging. BMC Geriatrics 2011, 11: 17

Supplemental Table 2. Full CARES survey questions

Recruitment Screening Log

B2. What is your date of birth? ____/____/____ (yyyy/mm/dd)

B3. What is your sex?
- Male
- Female

Enrollment Survey 1

B2. What is the highest grade of schooling you completed?
- No schooling
- Primary school
- Junior secondary school
- Senior/technical secondary school
- Junior college
- University or above
- Unknown
- Refused

B3. Are you currently….? [READ OPTIONS ALOUD]
- Married
- Separated/divorced
- Widowed
- Never married
- Unknown
- Refused

D7. How many days per week do you usually leave your house at any time of the day?
- 0 days
- 1 day
- 2 days
- 3 days
- 4 days
- 5 days
- 6 days
- 7 days

Enrollment Survey 2

A3. Enrollment City:
Suzhou
Yancheng

I. Functional Status Brief (Enrollment 2A Only) [1]		Can do alone without help	Sometimes need help	Often need help	Very often need help	All the time need help
I1a.	Get around from room to room in my house					
1b.	Stand up from sitting in a chair					
I1c.	Get on and off a toilet					
I1d.	Get in and out of bed					
I1e.	Walking around outdoors or in my neighborhood					
I1f.	Shopping for groceries					
I1g.	Climb a flight of stairs					
I1h.	Bathe myself in a shower or bath tub					
I2a.	Dressing and undressing					
I2b.	Prepare my breakfast or lunch					
I2c.	Feed myself					
I2d.	Wash my face and hands					
I2e.	Wash my whole body by taking a shower or bath					
I2f.	Take care of my feet and toenails					
I2g.	Make it to the toilet without an accident, like losing control of urination or bowel movement					
I3a.	Wash and iron my clothes					
I3b.	Make the beds or change sheets					
I3c.	Do “light” housework like dusting or tidying up					
I3d.	Do “heavy” housework like mopping or vacuuming the floor					
I3e.	Taking medication on schedule					
I3f.	Pay bills and manage my money					
I3g.	Prepare dinner					

J1. Now I want you to think about your physical health overall. During the past month, so the last 30 days, on how many days were you physically sick, injured, or not in good physical health? [2]

__ __ days [If >0, continue to J1a]

J1a. On how many of these days, did you need to stay in bed for at least half the day?

__ __ days

J2. During the past 30 days, for about how many days have you felt sad, depressed, worried, or anxious? [3]

__ __ days

J3. During the past 30 days, for about how many days have you felt very healthy and full of energy? [4]

__ __ days

Functional Status Extended (Enrollment 2B Only) [5]		Can do alone without help, without difficulty	Can do alone without help with some difficulty	Can do alone without help with great difficulty	Sometimes need help	Often need help	Very often need help	All the time need help
J4a.	Get around from room to room in my house							
J4b.	Stand up from sitting in a chair							
J4c.	Get on and off a toilet							
J4d.	Get in and out of bed							
J4e.	Walking around outdoors or in my neighborhood							
J4f.	Shopping for groceries							
J4g.	Climb a flight of stairs							
J4h.	Bathe myself in a shower or bath tub							
J5a.	Dressing and undressing							
J5b.	Prepare my breakfast or lunch							
J5c.	Feed myself							
J5d.	Wash my face and hands							
J5e.	Wash my whole body by taking a shower or bath							
J5f.	Take care of my feet and toenails							
J5g.	Make it to the toilet without an accident, like losing control of urination or bowel movement							
J6a.	Wash and iron my clothes							
J6b.	Make the beds or change sheets							
J6c.	Do “light” housework like dusting or tidying up							
J6d.	Do “heavy” housework like mopping or vacuuming the floor							
J6e.	Taking medication on schedule							

J6f. Pay bills and manage my money

J6g. Prepare dinner

D1. Using these categories, what was your total personal annual income in the last 12 months?

<10,000 RMB
10,000-29,000 RMB
30,000-39,000 RMB
40,000-49,000 RMB
50,000-100,000 RMB
>100,000 RMB
Unknown
Refused

F1. Thinking back over the past month, would you say your overall health is…? [READ OPTIONS ALOUD]

Excellent
Very good
Good
Fair
Poor

H2. Do you take a medication prescribed by a doctor or healthcare professional?

Yes → *Go to Question H14a*
No → *Go to Question H15*
Unknown

H14a. How many different types of medication prescribed by a doctor do you take every day? [6]
[NOTE: This should be number of separate medications and not number of pills.]

__ __ (number of medications)

H2. In the past 12 months, have you fallen? By that I mean, have you unintentionally went from standing, sitting, or leaning over to fall on the ground or floor? [7] [8]

Yes → *Go to Question H16a*
No → *Go to Question H17*
Unknown

How often have you fallen in the last 12 months?

__ __ (number of falls)

H17b. Now, thinking back to just the past 2 years, how many times have you been hospitalized and stayed at least 1 night?

- 0 times → *Go to Question H18*
- 1 time
- 2 times
- 3 times
- 4 times
- 5 or more times

References

[1] The functional status items in the following grid and featured on cards 1-3 are the same for both versions; version 2 includes an additional rating scale for all items.

[2] From US BRFSS 2014 Questionnaire

[3] From US BRFSS 2014 Questionnaire, but combines two mental health items.

[4] From US BRFSS 2014 Questionnaire

[5] This extended version incorporates scaling of difficulty for activities that can be done independently but still may be a challenge for the older person. This reflects scaling by Groningen Activity Restriction Scale (GARS) and includes all the items in this scale plus overlapping items with other prioritized indices

[6] Contributes to Groningen Frailty Indicator

[7] From US BRFSS 2014 with minor wording changes

[8] From US BRFSS 2014 with minor wording changes

Supplement Table 3. Sample characteristics in CARES, RETAIN, and PIVOT cohorts

Sample	CARES		CARES		RETAIN		PIVOT	
Scale	ADL-IADL-HELP-20		ADL-IADL-DIFFICULTY-20		ADL-IADL-DIFFICULTY-9		ADL-IADL-DIFFICULTY-9	
	748	(%)	758	(%)	404	(%)	1854	(%)
Age [a]								
60 to 64	122	(16)	120	(16)	NM		NM	
65 to 69	154	(21)	142	(19)	NM		795	(43)
70 to 74	148	(20)	153	(20)	210	(52)	507	(27)
75 to 79	105	(14)	104	(14)	194	(48)	379	(20)
80 to 84	173	(23)	185	(24)	NM		173	(9)
85 to 89	46	(6)	54	(7)	NM		NM	
Mean (SD)	73.7	(7.7)	74.0	(7.8)				
Sex								
Male	334	(45)	337	(44)	266	(66)	726	(39)
Female	414	(55)	421	(56)	138	(34)	1128	(61)
Study site								
Suzhou	379	(51)	375	(49)	NM		NM	
Yancheng	369	(49)	383	(51)	NM		NM	
Marital status [b]								
Not married	237	(32)	254	(33)	82	(20)	515	(28)
Married	511	(68)	504	(67)	322	(80)	1339	(72)
Educational attainment								
Secondary schooling or above	115	(15)	114	(15)	250	(62)	1069	(58)
Other	633	(85)	644	(85)	154	(38)	785	(42)
Personal annual income (RMB)								
<10,000	499	(67)	523	(69)	NM		NM	
10,000 to >100,000	224	(30)	203	(27)	NM		NM	
Unknown/Refused	25	(3)	32	(4)	NM		NM	
Self-reported health								
Poor-Fair	157	(21)	212	(28)	238	(45)	969	(52)
Good	284	(38)	294	(39)	109	(27)	566	(31)
Very Good-Excellent	307	(41)	252	(33)	57	(14)	319	(17)
History of falling [c]								
Yes	67	(9)	71	(9)	NM		NM	
No	680	(91)	687	(91)	NM		NM	
Chronic conditions								
0	261	(35)	286	(38)	NM		532	(29)
1	292	(39)	279	(37)	NM		635	(34)
2+	195	(26)	193	(25)	NM		687	(37)
Mean (SD)	1.0	(1.0)	1.0	(1.0)			1.3	(1.1)
History of hospitalization								
0	628	(84)	642	(85)	NM		NM	
1	93	(12)	87	(11)	NM		NM	
2+	27	(4)	29	(4)	NM		NM	
Number of medications per day								
0	364	(49)	390	(51)	NM		NM	
1	180	(24)	182	(24)	NM		NM	
2+	204	(27)	186	(25)	NM		NM	
Mean (SD)	0.9	(1.2)	0.9	(1.2)				
Mini mental state exam score								
Normal (25-30)	464	(62)	482	(64)	NM		NM	
Mild/Early (20-24)	225	(30)	205	(27)	NM		NM	

Moderate (10-19)	59	(8)	71	(8)	NM		NM	
Mean (SD)	25.0	(3.6)	24.9	(3.7)				
Number of days sick								
0	NM		599	(74)	299	(74)	1404	(76)
1 to 14	NM		156	(21)	74	(18)	356	(19)
15 to 30	NM		43	(6)	31	(8)	94	(5)
Mean (SD)			2.5	(6.0)	3.0	(7.5)	2.2	(5.9)
Number of days sick in bed								
0	NM		623	(82)	NM		1700	(92)
1 to 14	NM		124	(16)	NM		114	(6)
15 to 30	NM		10	(1)	NM		40	(2)
Mean (SD)			0.7	(2.9)			0.7	(3.6)
Number of days healthy								
0	NM		125	(16)	59	(15)	356	(19)
1 to 14	NM		57	(8)	16	(4)	94	(5)
15 to 30	NM		576	(76)	121	(30)	1404	(76)
Missing					208	(51)		
Mean (SD)			20.1	(11.0)	17.1	(13.2)	21.5	(13.7)
Number of days leaving home								
0	10	(1)	11	(1)	NM		4	(0)
1 to 6	78	(10)	73	(10)	NM		264	(14)
7	660	(88)	674	(89)	NM		1596	(86)
Mean (SD)	6.5	(1.5)	6.6	(1.4)			6.6	(1.2)

NM indicates that variable was not measured in sample

[a] Ages for enrolled participants were ≥60 years for CARES, 70-79 for RETAIN, and 65 to 82 years for PIVOT

[b] A few participants refused to report or were uncertain of current marital status (1 in CARES, 2 in RETAIN, and 6 in PIVOT); these were coded as not married.

[c] One participant did not know falling history in the CARES study ADL-IADL-HELP-20 subset and is imputed as having not fallen.

Supplement Table 4. Measurement indicators and concurrent validity correlations for ADL-IADL-HELP-20 and ADL-IADL-DIFFICULTY-20 scales

Scale	ADL-IADL-HELP-20		ADL-IADL-DIFFICULTY-20	
Response options	Requiring help		Requiring help or experiencing difficulty	
N	748		758	
**Measurement Indicators**				
Cronbach's Alpha [a]	0.96 (Excellent)		0.95 (Excellent)	
Invariant Item Ordering (H^T) [b]	0.50 (High)		0.45 (Medium)	
**Correlation to Scale Total** [c]				
Age	**0.22**	**(0.15, 0.29)**	**0.30**	**(0.23, 0.36)**
Self-rated health	**-0.27**	**(-0.34, -0.20)**	**-0.27**	**(-0.34, -0.21)**
Number of falls (prior year)	0.00	(-0.07, 0.08)	**0.16**	**(0.09, 0.23)**
Hospitalizations (in 2 years)	**0.13**	**(0.06, 0.20)**	0.04	(-0.03, 0.11)
Medications per day	**0.16**	**(0.09, 0.23)**	0.07	(-0.00, 0.14)
Chronic conditions	**0.11**	**(0.03, 0.18)**	**0.15**	**(0.08, 0.22)**
Mini mental state exam score	**-0.21**	**(-0.28, -0.15)**	**-0.27**	**(-0.33, -0.20)**
Sick days (in past month)	NM		**0.19**	**(0.12, 0.26)**
Sick in bed days (month)	NM		**0.28**	**(0.21, 0.34)**
Healthy days (month)	NM		**-0.09**	**(-0.16, -0.02)**
Days leaving home (in prior week)	**-0.25**	**(-0.31, -0.18)**	**-0.30**	**(-0.36, -0.23)**

NM indicates that variable was not measured in sample

Pearson correlations performed using the summed score of all item responses

[a] Categorical descriptions are based on previously published conventions for Cronbach's alpha

[a] Using previously establised conventions for H^T, items marked for good performance when mean Invariant Item Ordering (IIO) score is ≥0.50 and flagged for poor performance when IIO score is ≤0.10

[c] All scale and item scores were log-transformed prior to assessing correlations given the skewed nature of almost all distributions

Supplement Table 5. Item performance results for three item performance indicators and three concurrent validity indicators

				Item Performance						Concurrent Validity Correlations [c]					
**Scale Item**		**Domain**	**Subscale**	**Experiencing Difficulty [a]**		**Requiring Help [a]**		**Correlated Residuals [b]**		**Age**		**Self-rated Health**		**Days Leaving Home**	
**Good**															
5	Walking around outdoors or in my neighborhood	ADL	Ambulating	3.2%		1.8%	x	3	√	0.18		-0.22	√	-0.27	√
6	Shopping for groceries	IADL	Shopping	4.2%	√	5.1%	√	3	√	0.28	√	-0.25	√	-0.27	√
7	Climb a flight of stairs	ADL	Ambulating	12.7%	√	8.3%	√	6		0.35	√	-0.32	√	-0.27	√
8	Bathe myself in a shower or bath tub	ADL	Personal Hygiene	4.9%	√	3.6%		3	√	0.24	√	-0.26	√	-0.31	√
10	Prepare my breakfast or lunch	IADL	Food Preparation	1.5%	x	3.8%		3	√	0.22	√	-0.17		-0.21	√
13	Wash my whole body by taking a shower or bath	ADL	Personal Hygiene	4.1%	√	3.4%		3	√	0.23	√	-0.23	√	-0.25	√
14	Take care of my feet and toenails	ADL	Personal Hygiene	5.0%	√	6.3%	√	5		0.30	√	-0.24	√	-0.23	√
16	Wash and iron my clothes	IADL	Laundry	4.6%	√	6.2%	√	7		0.22	√	-0.19		-0.18	
17	Make the bed or change sheets	IADL	Housekeeping	4.6%	√	4.5%	√	4		0.27	√	-0.22	√	-0.22	√
18	Do "light" housework like dusting or tidying up	IADL	Housekeeping	4.1%	√	3.4%		4		0.24	√	-0.17		-0.24	√
19	Do "heavy" housework like mopping or vacuuming	IADL	Housekeeping	15.7%	√	9.4%	√	7		0.37	√	-0.29	√	-0.22	√
20	Prepare dinner	IADL	Food Preparation	2.8%		5.4%	√	7		0.25	√	-0.16		-0.23	√
**Neutral**															
1	Get around from room to room in my house	ADL	Ambulating	2.1%		0.8%	x	6		0.14		-0.18		-0.29	√
2	Stand up from sitting in a chair	ADL	Ambulating	3.2%		1.1%	x	4		0.16		-0.19		-0.29	√
3	Get on and off a toilet	ADL	Ambulating	2.1%		0.8%	x	6		0.14		-0.18		-0.29	√
4	Get in and out of bed	ADL	Ambulating	2.5%		0.7%	x	6		0.14		-0.17		-0.29	√
**Poor**															
9	Dressing and undressing	ADL	Dressing	1.7%	x	1.2%	x	6		0.14		-0.17		-0.22	√
11	Feed myself	ADL	Feeding	0.7%	x	0.4%	x	9	x	0.03	x	-0.09	x	-0.07	x
12	Wash my face and hands	ADL	Personal Hygiene	0.5%	x	0.7%	x	6		0.10		-0.14		-0.18	
15	Make it to the toilet without an accident	ADL	Continence	1.2%	x	1.7%	x	12	x	0.10		-0.20	√	-0.08	x

Items marked for good performance with a check mark and flagged for poor performance using an "x"

Items with ≥3 indicators flagged for poor performance are considered poor performing items

Items included in the 9-item Scale (ADL-IADL-DIFFICULTY-9) are highlighted green

[a] Items marked for good performance when percent of responses is ≥4% and flagged for poor performance when percent of responses is ≤2%

[b] Items marked for good performance when number of correlated residuals is < 1 standard deviation below median and flagged for poor performance when > 1 standard deviation above median

[c] Items are log-transformed and marked for good performance when the magnitude of correlation is ≥0.20 (moderately low correlation) and flagged for poor performance when the magnitude of correlation is ≤0.10 (low correlation)
